# Editorial:The Golden Era: Gold Nanomaterials for Bioapplications

**DOI:** 10.3389/fchem.2020.00780

**Published:** 2020-10-16

**Authors:** Shengyang Yang, Chen Zhou, Jinbin Liu

**Affiliations:** ^1^Department of Chemistry and Chemical Engineering, Yangzhou University, Yangzhou, China; ^2^School of Natural Science, University of Central Missouri, Warrensburg, MO, United States; ^3^School of Chemistry and Chemical Engineering, South China University of Technology, Guangzhou, China

**Keywords:** gold nanomaterials, bioapplication, sensor, therapy, device

The Organization for Economic Cooperation and Development defines biotechnology as the “application of science and technology to living organisms as well as parts, products, and models thereof, to alter living or non-living materials for the production of knowledge, goods, and services.” While the widespread potential of nanomaterials offered a key driving force for their research and development in the biotechnology field, the role played by many nanomaterials is still unfortunately limited today due to their hazardous properties. Gold nanomaterials often exhibit fascinating material characterizations and biofriendly properties that truly stand out from many other nanostructures; as a result, they have been extensively investigated for different bioapplications, including detection, imaging, diagnosis, drug delivery, and therapy. For their applications in various bioenvironments and aims, gold can be fine-tuned into many different nanostructures ranging from clusters and nanoparticles to layer membranes and complex microstructures. With the diverse biofunctions achieved from these structures, components, and surface chemistry, gold nanomaterials are attracting great attention from researchers to further explore their bioapplications.

In this Research Topic, we have collected three original research articles and one review written by 27 authors. These papers cover the important bioapplications of gold nanomaterials, including the stability of gold nanoparticles in a simulated tissue environment, protein and bacterial detection, infection therapy, the metabolic process of gold nanoparticles, and *in vivo* fluorescent temperature mapping. For example, Wu et al. reported the effect of BSA as a stabilizer of AuNPs and obtained AuNP/BSA conjugates with high colloidal stability against salt, acid, and base. They also discovered the non-thiolated DNA cannot replace proteins on the gold nanoparticles, but thiolated DNA can be controllably attached to AuNP/BSA conjugates under certain conditions. This work not only reveals interesting biointerfaces on AuNPs for DNA and proteins absorption, but also provides a controllable method to obtain stable AuNP/BSA/DNA conjugates using a protein corona as a stabilizer. Chen et al. employed ligand exchange and interfacial assembly methods to fabricate plasmonic superlattice membranes, which possessed high structural homogeneity and exhibited near-field confined hotspots uniformly fashioned over the whole substrate, enabling ultrasensitive SERS-based detection of trace chemicals and label-free direct detection of BSA. The work represents a significant step toward the exploitation of plasmonic membranes for a plethora of technical applications such as the next-generation of nanophotonics devices and point-of-care biosensors. Hua et al. synthesized renal clearable gold nanoparticles and embedded them into silk to obtain a gold nanoparticle-functionalized silk film for *in vivo* fluorescent temperature mapping. The renal clearance property of gold nanoparticles together with biodegradable silk significantly minimized the potential toxicity and side effects of the hybrid material, which is an important step for the future development of implantable and wearable sensor devices. Tang et al. provided an overview of research progress on the development of versatile gold nanoclusters (AuNCs) for bacterial detection and infection treatment, and highlighted the mechanisms of action of designed diagnostic and therapeutic agents.

In summary, the goal of our Research Topic is to emphasize the structure design, surface ligand tailoring, composite assembly, and bioapplications of gold nanomaterials. The functions of gold nanomaterials generally depend on their size, nanostructures, and surface chemistry, which can be plentifully regulated to extend their bioapplications and stride forward with their clinic translation in the future. Admittedly, the limited number of studies presented in this Research Topic cannot cover the full scope of gold nanomaterials and their bioapplications; however, we sincerely hope the papers we collected in this special edition can be beneficial to your research endeavor.

## Author Contributions

All authors listed have made a substantial, direct and intellectual contribution to the work, and approved it for publication.

## Conflict of Interest

The authors declare that the research was conducted in the absence of any commercial or financial relationships that could be construed as a potential conflict of interest.

